# Thermal processing and geographical origin as drivers of terpenoids variation in *Anethum graveolens* L. essential oils: A biplot analysis

**DOI:** 10.1371/journal.pone.0327018

**Published:** 2025-08-01

**Authors:** Karim Farmanpour Kalalagh, Mehdi Mohebodini, Naser Sabaghnia, Arman Beyraghdar Kashkooli, Hassan Esmaeili

**Affiliations:** 1 Department of Horticultural Science, Faculty of Agriculture, Tarbiat Modares University, Tehran, Iran; 2 Department of Horticultural Science, Faculty of Agricultural Science and Natural Resources, University of Mohaghegh Ardabili, Ardabil, Iran; 3 Department of Plant Production and Genetics, Faculty of Agriculture, University of Maragheh, Maragheh, Iran; 4 Department of Agriculture, Medicinal Plants and Drugs Research Institute, Shahid Beheshti University, 1983969411, Tehran, Iran; University of Kashan, IRAN, ISLAMIC REPUBLIC OF

## Abstract

Dill (*Anethum graveolens* L.), a medicinal-vegetable plant renowned for its aromatic and functional properties, exhibits significant variation in essential oil composition due to geographical origin (genotypic diversity) and post-harvest drying temperatures (DTs). This study aimed to (1) quantify the effects of geographical origin (as a proxy for genotype) and DTs on essential oil yield and composition, and (2) evaluate how specific genotypes respond to thermal processing. Six *A. graveolens* genotypes from distinct Iranian regions (Mashhad, Ardabil, Parsabad, Bushehr, Esfahan, and Kerman) were cultivated under uniform field conditions in Ardabil, Iran, to isolate genotypic variation. Post-harvest treatments included environmental shade drying and oven drying at 40°C and 60°C, creating unique combinations of genotype-treatment (CGT). Using CGT × character biplot analysis, we assessed interactions between genotype, DT, and essential oil compositions. The results revealed significant CGT-driven variation: shade drying enhanced α-Phellandrene levels in Kerman and Esfahan genotypes (57.49% and 55.51%), while oven drying at 40°C maximized Myristicin content (1.72%) in the Ardabil genotype and essential oil yield in Parsabad (1.86% w/v). High-temperature drying (60°C) reduced essential oil content in sensitive genotypes. β-Pinene and γ-Terpinene emerged as discriminative markers for genotype performance. Critically, the Parsabad genotype at 40°C and the Ardabil genotype demonstrated superior essential oil yields, whereas genotype-specific responses to DT highlighted the need for tailored post-harvest protocols. This study establishes CGT interactions as pivotal drivers of *A. graveolens* essential oil chemotypes, offering actionable strategies for genotype-specific drying protocols to optimize industrial production and breeding programs.

## 1. Introduction

Public health priorities and environmental sustainability concerns are reshaping global demand for nutritious and safe food products. Medicinal-edible plants, which serve dual roles in dietary and therapeutic applications, are central to this transition due to their direct impact on human well-being. Dill (*Anethum graveolens* L.), an aromatic herb from the Apiaceae family, originates from southwestern and central Asia and is now cultivated globally, including in southeastern Europe, Asia, and the United States. In Iran, it grows naturally in the northeastern, northwestern, and central regions. *A. graveolens* is valued for its diverse biochemical utility; its seeds and flowers are rich in essential oils, fatty acids, phenolic acids, and flavonoids, while stems and leaves contain lower essential oil concentrations [1]. The essential oil’s main constituents, α-phellandrene and germacrene D, vary based on drying temperature (DT), cultivar type, seasonal dynamics, extraction methods, etc. influencing the oil’s biological properties [2]. *A. graveolens* essential oil contains bioactive compounds such as α-phellandrene [3,4], Myristicin [5,6], α-Pinene [7,8], β-Pinene [9,10], β-Myrcene [11,12], α-Terpinene [13,14], Sabinene [15,16], Camphene [17], etc., which exhibit significant biological properties. These attributes indicate its potential for versatile applications in pharmaceuticals and the food industry. However, the therapeutic and commercial utilization of these compounds requires careful consideration of toxicity thresholds, dosage optimization, and adherence to safety protocols. For instance, myristicin, while bioactive, demonstrates neurotoxic potential at elevated concentrations [18], necessitating rigorous toxicological evaluations to establish safe exposure limits.. Research shows that the chemical composition and functional characteristics of *A. graveolens* essential oil are influenced by geographical origin and DTs. Drying is a critical post-harvest step, significantly altering the essential oil’s chemical makeup and functional attributes [19]. DT affects the concentration and bioavailability of specific metabolites. For instance, higher DTs decrease essential oil yield in *Mentha piperita* [20], while sun-drying and specific oven-DTs optimize essential oil content in other plants [21]. These findings highlight the importance of optimal drying techniques to preserve essential oil integrity and bioactivity.

Given this impact, it’s crucial to study *A. graveolens* from various provinces in Iran. While global studies have explored essential oil variability [22,23], there’s limited research on how DTs affect *A. graveolens* essential oil composition under Iranian climate conditions. Understanding these effects is vital for optimizing post-harvest processing and enhancing the medicinal and commercial value of *A. graveolens*. This study examines the interaction between genetic and environmental factors by evaluating the combined effect of genotype and treatment—specifically, the DT applied to each genotype—on essential oil yield, quality, and composition. This interaction, referred to as the genotype-treatment (G × T) combination, provides insights into how intrinsic (genetic) and extrinsic (environmental or post-harvest) variables influence plant secondary metabolite profiles. To analyze these complex interactions, a combination of genotype-treatment (CGT) × character biplot analysis was employed, offering a multivariate framework to identify optimal genotype-treatment combinations for maximizing essential oil characteristics..

## 2. Materials and methods

### 2.1. Seed collection, planting, growing, and harvesting

This study investigates six regional dill (*Anethum graveolens*) genotypes from various parts of Iran (Table 1). The genotypes analyzed included Mashhad from the northeast, Ardabil and Parsabad from the northwest, Bushehr from the south, and Esfahan and Kerman from the central regions. The research was conducted in a field in Ardabil, Iran, with loamy soil composed of 14% clay, 43% sand, and 43% silt. The area has an average annual temperature of 9°C, with maximum and minimum averages of 15°C and 3°C, respectively. The region receives an average annual precipitation of 303 mm and has a relative humidity of 70%. Seeds of six genotypes were collected and assessed for seed vigor before being sown in three blocks, arranged in a completely randomized block design. The plants were irrigated twice weekly, and the leaves and flowers were harvested at full bloom.

**Table 1:** Name and geographic coordinates of dill (*Anethum graveolens* L.) genotypes in this study.

_Name_ ^Ge-Co^	Altitude (m)	Latitude (N)	Longitude (E)	Rainfall (mm)	Temp. (°C)
Ardabil	1,348	38°15′13″	48°17′56″	295.5	8.5
Bushehr	10	28°54′43″	50°49′09″	268.1	25.1
Esfahan	1,580	32°39′22″	51°40′19″	130.3	16.3
Mashhad	982	36°15′56″	59°36′39″	250.7	15.7
Kerman	1,764	30°16′53″	57°05′11″	140.6	15.8
Parsabad	44	39°39′06″	47°55′13″	550.1	18.2
Planting field	1384	38°12’40“	48°17’37“	295.5	8.5

Ge-Co: Geographic coordinates.

### 2.2. Thermal processing and essential oil extraction

To examine the effects of different drying temperatures (DTs) on the aroma profile of various genotypes, fresh leaves and flowers underwent three DTs prior to essential oil extraction: shade drying, and oven drying at 40°C and 60°C. For shade drying, fresh aerial parts from each block and each genotype were spread on a cotton-covered surface in a temperature-controlled drying chamber with active airflow to ensure consistent ventilation, avoiding direct sunlight. Drying continued until plant moisture content reached 10–12%. For oven drying at 40°C and 60°C, samples were processed in a forced-air convection oven (OD 53 Model, Irankhodsaz company) until the same moisture threshold (10–12%) was achieved. Post-drying, 50 grams of dried plant material per genotype was subjected to hydrodistillation using a Clevenger apparatus for 4 hours. The resultant essential oil was separated, dehydrated with anhydrous sodium sulfate, and stored in amber glass vials at 4°C until chromatographic analysis..

### 2.3. Gas Chromatography-Mass Spectrometry (GC-MS) analysis and components identification

The chemical composition of the essential oils was analyzed using a GC-MS system (Agilent 5977A Series MSD) and quantified via a GC-FID system (Agilent 7890 B series). Both instruments were fitted with an HP-5MS capillary column (30 m length × 0.25 mm inner diameter × 0.25 μm film thickness). Helium was used as the carrier gas at a flow rate of 1 ml/min with a split ratio of 1:50. The components of the essential oils were identified based on their retention times (RTs) and indices (RIs), utilizing NIST05 and Wiley7 mass spectral libraries. A homologous series of *n*-alkanes was employed to calculate RIs. The constituents were further confirmed by comparing the RIs with reference spectra from the Adams database [24] and the National Institute of Standards and Technology (NIST) Chemistry WebBook (https://webbook.nist.gov).

### 2.4. Statistical analysis

The key components analyzed included: α-Thujene (A-THU), Camphene (CAM), Sabinene (SAB), β-Pinene (B-PIN), β-Myrcene (B-MYR), α-Phellandrene (A-PH), α-Terpinene (A-TER), *p*-Cymene (R-CY), β-Phellandrene (B-PH), γ-Terpinene (G-TE), α-Terpinolene (A-T), Sabinol (Sab), Dill ether (Dill), Carvacrol (CAR), Germacrene D (GER), Myristicin (MYR), Dillapiole (DIL), Neophytadiene (NEO), Hexahydrofarnesyl acetone (HEX), and essential oil yield (EO).

The normality of the obtained dataset was assessed using the Anderson-Darling procedure (Minitab, Pennsylvania, USA). Principal component analysis (PCA) was conducted utilizing a combination genotype-treatment (CGT) × character biplot, with the model applied using the GGEbiplot software version testBiplotxlsx (https://ggebiplot.com/). The following equation was employed:


$$\frac{a_{ij}-b_{j}}{S_{j}}=\sum_{n=1}^{2}\lambda_{n}\xi_{in}\eta_{jn}+\varepsilon_{ij}$$

where a_ij_ is the score of the combination genotype-treatment (CGT) i for character j, b_j_ is the average of CGT in character j, S_j_ is the square root of variance for character j, λ_n_ is the singular value for PCn, ξ_in_ and η_in_ are values for CGT i and character j on PCn, respectively, and ε_ij_ is the model’s residual. To achieve symmetric scores for characters as testers and CGTs as entries, the eigenvalues were adjusted through vector absorption, enabling a more interpretable visualization of the interrelationships among CGTs, characters, and their interactions. A scaling factor of 1 was applied to obtain a rescaled dataset, while a centering value of 2 was used to centralize the dataset. Singular value decomposition (SVP) was performed with a value of 2 to calculate the singular values. Lastly, SVP with a value of 1 was implemented to derive singular amounts suitable for visually interpreting associations among CGTs via generating their vectors from plat center.

### 2.5. Ethics declaration

As this research exclusively involved plant materials and analytical procedures, no ethics committee review was required. All cultivation protocols and laboratory analyses adhered to institutional and national regulatory standards, with authors affirming adherence to established ethical norms and research integrity guidelines.

## 3. Results

### 3.1. Biplot analysis of genotype-treatment interactions

**Fig 1:**
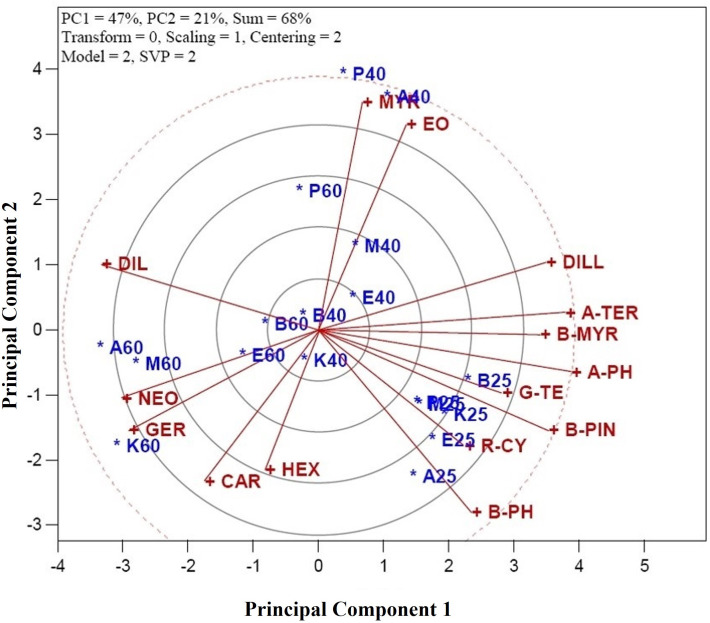
Associations among characters of dill (*Anethum graveolens* L.) genotypes under various drying temperatures. Characters are: α-Thujene (A-THU), Camphene (CAM), Sabinene (SAB), β-Pinene (B-PIN), β-Myrcene (B-MYR), α-Phellandrene (A-PH), α-Terpinene (A-TER), *p*-Cymene (R-CY), β-Phellandrene (B-PH), γ-Terpinene (G-TE), α-Terpinolene **(A-T)**, Sabinol (SAB), Dill ether (DILL), Carvacrol (CAR), Germacrene D (GER), Myristicin (MYR), Dillapiole (DIL), Neophytadiene (NEO), Hexahydrofarnesyl acetone (HEX), and essential oil yield (EO). Combination genotype-treatment (CGT) are: A25 (genotype Ardabil + shade drying), A40 (genotype Ardabil + oven drying at 40°C), A60 (genotype Ardabil + oven drying at 60°C), B25 (genotype Bushehr + shade drying), A40 (genotype Bushehr + oven drying at 40°C), A60 (genotype Bushehr + oven drying at 60°C), E25 (genotype Esfahan + shade drying), E40 (genotype Esfahan + oven drying at 40°C), E60 (genotype Esfahan + oven drying at 60°C), M25 (genotype Mashhad + shade drying), M40 (genotype Mashhad + oven drying at 40°C), M60 (genotype Mashhad + oven drying at 60°C), K25 (genotype Kerman + shade drying), K40 (genotype Kerman + oven drying at 40°C), K60 (genotype Kerman + oven drying at 60°C), P25 (genotype Parsabad + shade drying), P40 (genotype Parsabad + oven drying at 40°C), and P60 (genotype Parsabad + oven drying at 60°C).

The genotype-treatment (CGT) × character interaction biplot model accounted for 68% of the variation within the standardized two-way dataset (Fig 1). This substantial proportion of variation reflects the relationships among measured traits (e.g., essential oil yield and composition) and treatments (shade drying, 40°C, and 60°C). Vectors originating from the biplot’s center were generated to visualize associations between traits and CGT combinations, enabling intuitive interpretation of additive and crossover interactions. SVP with a scaling factor of 1 was applied to optimize the biplot’s visual interpretation, ensuring accurate representation of CGT-trait associations. The model’s effectiveness in structuring the dataset was further validated by its capacity to rank CGTs based on trait performance, critical for identifying genotype-specific responses to drying protocols.

### 3.2. Character associations and interactions

In this study, the biplot model applied, as recommended by Yan [25], proved to be an effective tool for exploring and visualizing entry-by-tester relationships. The model captured substantial variability, providing a clearer understanding of how different genotype-treatment combinations influence character traits. The associations among these variables were further clarified by examining the cosine of the vectors: cos 0° = +1 (positive relation), cos 90° = 0 (no relation), and cos 180° = −1 (negative relation). These cosine values allowed for a nuanced understanding of characters interactions. Notable associations were observed between several pairs of characters. For instance, a positive association was found between Neophytadiene (NEO) and Germacrene D (GER), indicating that these compounds tend to vary together. Similarly, Hexahydrofarnesyl acetone (HEX) was positively associated with Carvacrol (CAR), and *p*-Cymene (R-CY) showed a positive relationship with β-Phellandrene (B-PH). Other positive associations included γ-Terpinene (G-TE) with β-Pinene (B-PIN), α-Terpinene (A-TER) with β-Myrcene (B-MYR), and Myristicin (MYR) with essential oil yield (EO). These relationships were illustrated through the close angles between their vectors, with cos 0° = +1 indicating strong associations. These findings align with previous research on *A. graveolens* [26], which reported positive interrelationships between *p*-Cymene and β-Phellandrene, as well as Germacrene D and Neophytadiene in *A. graveolens* genotypes. This consistency reinforces the reliability of the biplot model in detecting meaningful patterns and associations in the data. Overall, the use of the CGT × character interaction biplot model in this study provided valuable insights into the complex interactions between genotype-treatment combinations and the chemical compositions of *A. graveolens* genotypes.

In contrast, several pairs of characters exhibited near-zero associations, indicating weak or negligible interactions. For instance, the relationship between Myristicin and EO with α-Phellandrene (A-Ph) was nearly non-existent. Similarly, the associations between Dillapiole and Myristicin, and EO, as well as between Neophytadiene (NEO) and Germacrene D (GER) with *p*-Cymene (R-CY) and β-Phellandrene (B-PH), were minimal. These minimal associations were represented by vectors nearly perpendicular to each other in the biplot (Fig 1), suggesting little to no correlation between the respective compounds.

Moreover, strong negative correlations were identified between several character pairs, implying that an increase in one compound’s magnitude corresponded to a decrease in another’s concentration. For example, a negative correlation was observed between Myristicin and EO with Hexahydrofarnesyl acetone (HEX) and Carvacrol (CAR). Additionally, Dill ether (DIL) exhibited negative correlations with γ-Terpinene (G-TE) and β-Pinene (B-PIN), as well as with Neophytadiene (NEO) and Germacrene D (GER). These negative correlations were represented by obtuse angles between their vectors in the biplot (Fig 1), indicating an inverse relationship between these compounds. Such findings suggest that certain compounds may exert antagonistic effects on each other, which is important for understanding their roles in the overall chemical profile of *A. graveolens*. The biplot method provided a clear visual representation of the associations among characters in *A. graveolens*, enabling a deeper understanding of the relationships between various compounds.

However, these direct measurements may not always correspond exactly to the results of the biplot analysis. The biplot, by considering the multivariate relationships and interactions in the dataset, often reveals subtler patterns and associations not immediately apparent from pairwise correlation analysis. Thus, the use of the biplot method in this study allowed for a more holistic interpretation of the complex interactions within the chemical composition of *A. graveolens*, highlighting the importance of integrating both visual and quantitative analyses when studying multifaceted biological datasets.

### 3.3. Genotype-treatment performance and optimization

**Fig 2:**
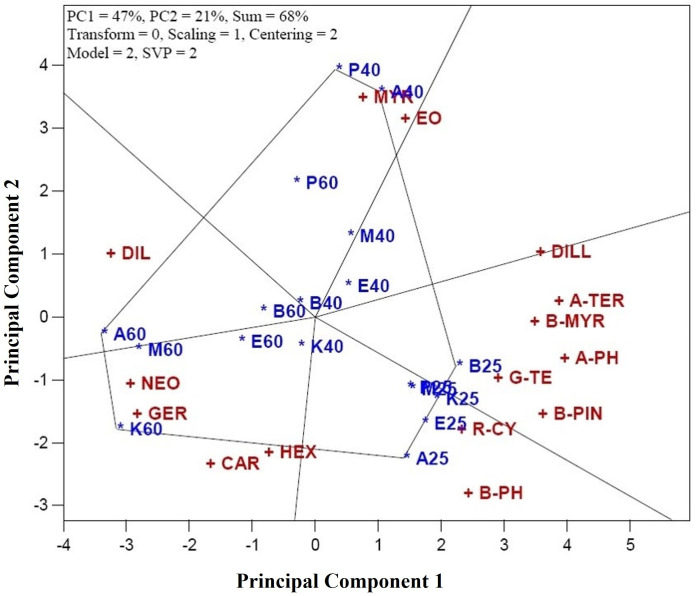
Identification of the best combination genotype-treatment (CGT) for characters of dill (*Anethum graveolens* L.). Characters are: α-Thujene (A-THU), Camphene (CAM), Sabinene (SAB), β-Pinene (B-PIN), β-Myrcene (B-MYR), α-Phellandrene (A-PH), α-Terpinene (A-TER), *p*-Cymene (R-CY), β-Phellandrene (B-PH), γ-Terpinene (G-TE), α-Terpinolene (A-T), Sabinol (SAB), Dill ether (DILL), Carvacrol (CAR), Germacrene D (GER), Myristicin (MYR), Dillapiole (DIL), Neophytadiene (NEO), Hexahydrofarnesyl acetone (HEX), and essential oil yield (EO). Combination genotype-treatment (CGT) are: A25 (genotype Ardabil + shade drying), A40 (genotype Ardabil + oven drying at 40°C), A60 (genotype Ardabil + oven drying at 60°C), B25 (genotype Bushehr + shade drying), A40 (genotype Bushehr + oven drying at 40°C), A60 (genotype Bushehr + oven drying at 60°C), E25 (genotype Esfahan + shade drying), E40 (genotype Esfahan + oven drying at 40°C), E60 (genotype Esfahan + oven drying at 60°C), M25 (genotype Mashhad + shade drying), M40 (genotype Mashhad + oven drying at 40°C), M60 (genotype Mashhad + oven drying at 60°C), K25 (genotype Kerman + shade drying), K40 (genotype Kerman + oven drying at 40°C), K60 (genotype Kerman + oven drying at 60°C), P25 (genotype Parsabad + shade drying), P40 (genotype Parsabad + oven drying at 40°C), and P60 (genotype Parsabad + oven drying at 60°C).

Fig 2 demonstrates how the biplot method facilitates the comparison of genotype-treatment (CGT) combinations based on the evaluated traits, enabling the superior performers in specific traits. These identified CGTs could serve as prime candidates for breeding programs or commercial release. To aid in comparing vertex CGTs, a hexagon was constructed with perpendiculars drawn to its sides, visually highlighting the top-performing genotype and DT combinations. The six key vertex CGTs identified in the analysis were: A25 (genotype Ardabil + shade drying), A40 (genotype Ardabil + oven drying at 40°C), A60 (genotype Ardabil + oven drying at 60°C), B25 (genotype Bushehr + shade drying), P40 (genotype Parsabad + oven drying at 40°C), and K60 (genotype Kerman + oven drying at 60°C). These vertex CGTs represent the optimal combinations for specific traits. Notably, A40 and A60 showed the highest values for Dill ether (DILL) and Dillapiole (DIL), respectively, with A40 exhibiting superior levels of DILL and A60 having the highest concentrations of DIL. The vertex CGT of P40 demonstrated the greatest amounts of myristicin (MYR) and EO, while A60 displayed the highest levels of *p*-Cymene (R-CY) and β-Phellandrene (B-PH). K60 showed the highest values for Neophytadiene (NEO), Germacrene D (GER), Carvacrol (CAR), and Hexahydrofarnesyl acetone (HEX). B25 performed exceptionally well in other characters, especially in terms of α-Phellandrene (A-PH), a cyclic monoterpene biosynthesized through the methylerythritol phosphate pathway in plastids, is known for its bioactive properties, including antifungal, anti-inflammatory, wound-healing, tissue damage modulation, oxidative stress reduction, and TNF-α regulation [27]. The high concentration of this component in genotype Bushehr under shade drying (B25) suggests the particular benefit of shade drying for enhancing A-PH levels in this genotype. Conversely, the performance of other CGTs was lower. For example, the P60’s performance was inferior to P40 in terms of Myristicin and EO content. Myristicin, a phenylpropanoid, has anti-inflammatory, anti-intestinal, and apoptosis-protective effects [6].

The study suggests prioritizing the genotype Parsabad combined with oven drying at 40°C (P40) to achieve high amounts of Myristicin and EO. On the other hand, shade drying of genotypes Ardabil and Bushehr (A25 and B25) are recommended for higher concentrations of most components, while higher DTs (e.g., 60°C) for oven drying are beneficial for increasing DIL, NEO, GER, CAR, and HEX concentrations in genotypes Ardabil and Kerman (A60 and K60).

### 3.4. Ideal CGT proximity and implications

**Fig 3:**
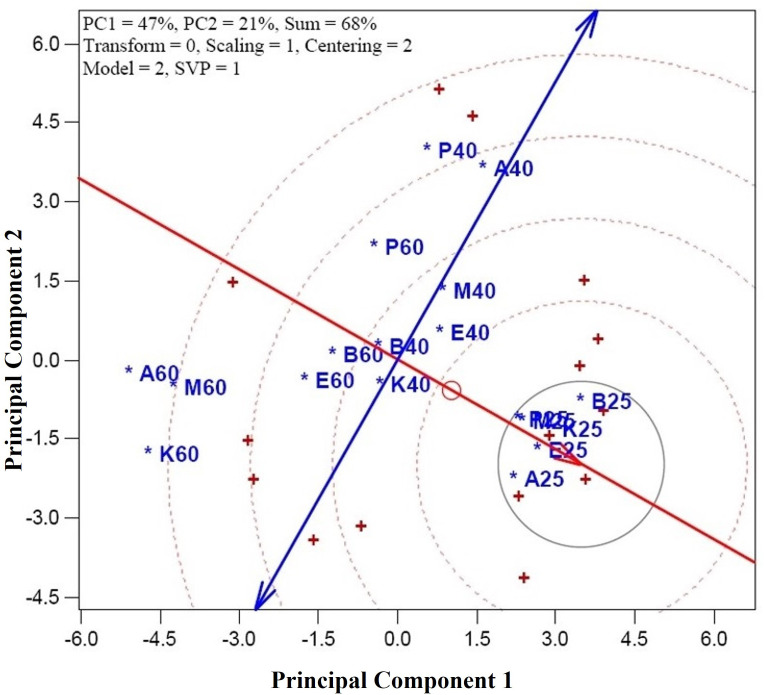
The perfect entry or combination genotype-treatment (CGT) of dill (*Anethum graveolens* L.). CGTs are: A25 (genotype Ardabil + shade drying), A40 (genotype Ardabil + oven drying at 40°C), A60 (genotype Ardabil + oven drying at 60°C), B25 (genotype Bushehr + shade drying), A40 (genotype Bushehr + oven drying at 40°C), A60 (genotype Bushehr + oven drying at 60°C), E25 (genotype Esfahan + shade drying), E40 (genotype Esfahan + oven drying at 40°C), E60 (genotype Esfahan + oven drying at 60°C), M25 (genotype Mashhad + shade drying), M40 (genotype Mashhad + oven drying at 40°C), M60 (genotype Mashhad + oven drying at 60°C), K25 (genotype Kerman + shade drying), K40 (genotype Kerman + oven drying at 40°C), K60 (genotype Kerman + oven drying at 60°C), P25 (genotype Parsabad + shade drying), P40 (genotype Parsabad + oven drying at 40°C), and P60 (genotype Parsabad + oven drying at 60°C).

Fig 3 illustrates the ideal position for a perfect CGT, with the CGT combinations closest to this ideal position being considered the best-performing. Among the combinations studied, all genotypes under shade drying conditions (A25, B25, E25, K25, and M25) were positioned nearest to the ideal location on the graph, indicating their superior performance in terms of essential oil yield and quality characteristics. These genotypes have the potential to be optimal candidates for breeding programs or post-harvest management due to their proximity to the ideal CGT. Conversely, combinations positioned farthest from the ideal CGT, such as A60, K60, and M60, were less desirable. Treatments involving oven drying at higher DTs (60°C) demonstrated lower efficiency than shade-dried genotypes, suggesting that high-DT may be less optimal for maximizing essential oil quality and yield in these particular genotypes. For the remaining CGTs, the ranking was based on their proximity to the ideal CGT, with most positioned near the average axis, indicating moderate performance compared to the best-performing shade-dried genotypes. This implies that while they exhibited acceptable performance, they did not achieve the same high quality and yield as the shade-dried combinations. Therefore, using graphic multivariate techniques, like the one used in this study, is crucial for identifying the optimal genotype-treatment combinations that can lead to improved yield management and more effective breeding strategies for *A. graveolens*.

### 3.5. Discriminative potential of traits

**Fig 4:**
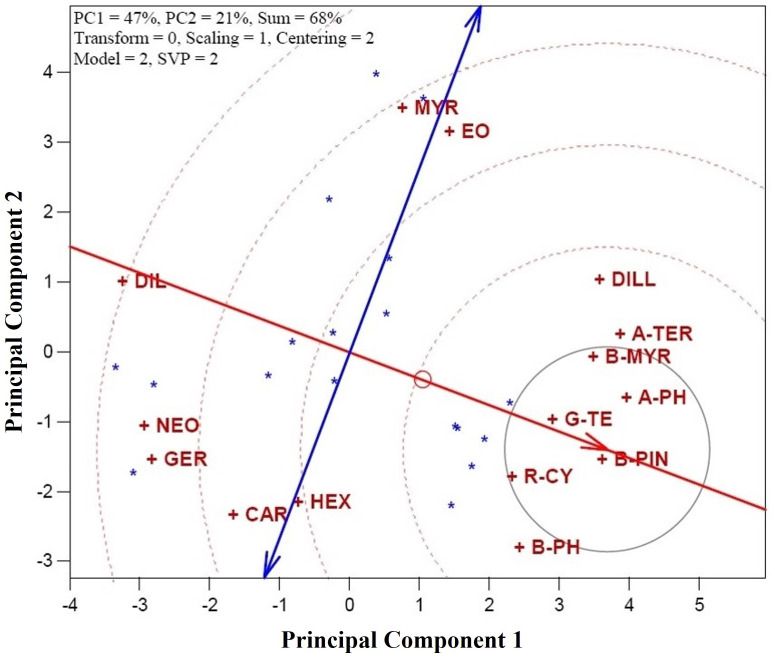
Identification of the perfect character of dill (*Anethum graveolens* L.). Characters are: α-Thujene (A-THU), Camphene (CAM), Sabinene (SAB), β-Pinene (B-PIN), β-Myrcene (B-MYR), α-Phellandrene (A-PH), α-Terpinene (A-TER), *p*-Cymene (R-CY), β-Phellandrene (B-PH), γ-Terpinene (G-TE), α-Terpinolene (A-T), Sabinol (SAB), Dill ether (DILL), Carvacrol (CAR), Germacrene D (GER), Myristicin (MYR), Dillapiole (DIL), Neophytadiene (NEO), Hexahydrofarnesyl acetone (HEX), and essential oil yield (EO).

The discriminative potential of a character is determined by the magnitude of its variability, with greater variation indicating a higher ability to distinguish among CGTs. This potential is reflected by the character’s proximity to the assumed location of the perfect character (Fig 4). Characters located closer to the perfect character exhibit more discrimination potential, meaning they are better at differentiating between CGTs. Conversely, characters positioned further away are less favorable due to their reduced ability to distinguish between CGTs. In this context, β-Pinene (B-PIN) emerges as the most discriminative character, followed by Terpinene (G-TE), α-Phellandrene (A-PH), *p*-Cymene (R-CY), and β-Myrcene (B-MYR). These characters demonstrate the highest discriminative potential, making them highly effective at differentiating among the *A. graveolens* genotypes. Most assessed characters show above-average discriminative potential due to their positions on the right-side of plot regarding vertical axis, making them useful for distinguishing between different genotypes and treatments in future studies (Fig 4). On the other hand, Dillapiole (DIL), followed by Neophytadiene (NEO) and Germacrene D (GER), were found to have low discriminative potential, making them less effective for distinguishing among genotypes and treatments and not recommended for inclusion in future investigations of *A. graveolens* essential oil profiles. The typical ability of a character, reflecting its symbolic importance, can be assessed by the angle between the character’s axis and the average character axis. Smaller angles indicate higher typical ability, meaning those characters are more representative of the dataset’s general trends. Characters with the smallest angles, such as B-PIN, G-TE, and A-PH, demonstrate greater typical ability and are highly representative of the dataset’s structure. In contrast, characters like DIL and B-PH have moderate angles, indicating their moderate typical ability. Finally, β-Myrcene (B-MYR), Carvacrol (CAR), Hexahydrofarnesyl acetone (HEX), and essential oilhave larger angles with the axis, reflecting their lower typical ability to represent the dataset’s overall structure. This analysis helps identify the most influential characters for discriminating between genotypes and treatments, which is crucial for selecting the best candidates in breeding and commercial applications of *A. graveolens*.

### 3.6. EO yield and genotype stability

**Fig 5:**
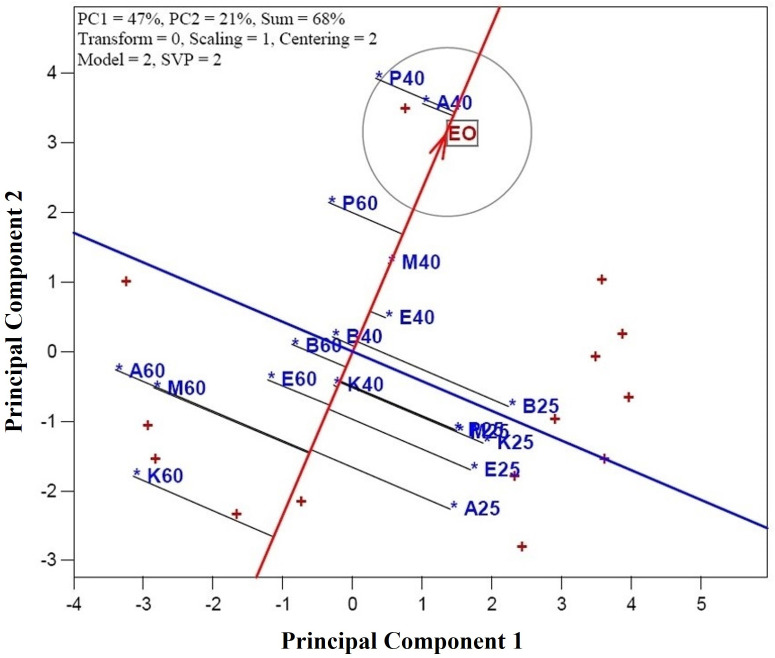
Testing the performance of various of combination genotype-treatment (CGT) in essential oil of dill (*Anethum graveolens* L.). CGTs are: A25 (genotype Ardabil + shade drying), A40 (genotype Ardabil + oven drying at 40°C), A60 (genotype Ardabil + oven drying at 60°C), B25 (genotype Bushehr + shade drying), A40 (genotype Bushehr + oven drying at 40°C), A60 (genotype Bushehr + oven drying at 60°C), E25 (genotype Esfahan + shade drying), E40 (genotype Esfahan + oven drying at 40°C), E60 (genotype Esfahan + oven drying at 60°C), M25 (genotype Mashhad + shade drying), M40 (genotype Mashhad + oven drying at 40°C), M60 (genotype Mashhad + oven drying at 60°C), K25 (genotype Kerman + shade drying), K40 (genotype Kerman + oven drying at 40°C), K60 (genotype Kerman + oven drying at 60°C), P25 (genotype Parsabad + shade drying), P40 (genotype Parsabad + oven drying at 40°C), and P60 (genotype Parsabad + oven drying at 60°C).

Fig 5 evaluates the performance *A. graveolens* genotypes across different DTs (CGT) in terms of essential oil content. The horizontal axis represents essential oil, with an arrow indicating its direction. Genotypes A40 (genotype Ardabil + oven drying at 40°C) and P40 (genotype Parsabad + oven drying at 40°C) were identified as the most desirable for achieving high essential oil yields, while K60 (genotype Kerman + oven drying at 60°C) was the least favorable CGT for essential oilproduction (Fig 5). Additionally, genotypes P60 (genotype Parsabad + oven drying at 60°C), M40 (genotype Mashhad + oven drying at 40°C), E40 (genotype Esfahan + oven drying at 40°C), B40 (genotype Bushehr + oven drying at 40°C), and B25 (genotype Bushehr + shade drying) demonstrated above-average essential oil performance, as they are positioned above the average axis. The distance of each genotype from the horizontal axis is indicative its variability, with smaller distances suggesting lower variation, which is preferable for selection as it reflects more consistent performance. In this regard, A40 (genotype Ardabil + oven drying at 40°C) stands out due to its low variation. In contrast, A60 (genotype Ardabil + oven drying at 60°C), with its lower essential oil yield and greater distance from the horizontal axis, exhibits higher variability and is considered one of the least favorable CGTs.

## 4. Discussion

The research investigated the influence of geographical origin and varying drying temperatures (DTs) on the yield and quantity and quality of essential oil profile in *A. graveolens* genotypes from different regions of Iran. This study offers new perspectives on the complex relationships between genotype, DTs, and essential oil composition, highlighting the importance of managing environmental and post-harvest factors to enhance essential oil production in medicinal plants. The six *A. graveolens* genotypes examined, originating from distinct regions of Iran (Mashhad, Ardabil, Parsabad, Bushehr, Esfahan, and Kerman), showed significant variation in essential oil content and its components. This finding aligns with previous studies emphasizing the impact of geographical factors on the chemical profile of essential oils in various medicinal plants. For instance, researchers reported high genetic diversity in *A. graveolens* genotypes, with notable differences in essential oil composition based on the cultivation region [28,29]. Similar results have been observed in other plants such as oregano, basil, and lavender, where environmental factors like soil type, climate, and water availability significantly influence the chemical composition of essential oils [30]. In the present study, *A. graveolens* genotypes from different regions exhibited variability in the concentrations of key compounds such as β-Pinene, α-Phellandrene, Dillapiole, Myristicin, and Carvacrol. These differences were mainly due to the genetic backup of the genotypes and the environmental conditions specific to each region. For example, genotypes from Ardabil and Bushehr, when shade-dried, showed high levels of α-Phellandrene, known for its antifungal and anti-inflammatory properties [27]. Conversely, genotypes from Parsabad, subjected to oven drying at 40°C, yielded higher amounts of Myristicin, a phenylpropanoid compound with anti-inflammatory and anti-cancer properties [6]. The findings suggest that selecting genotypes based on their geographic origin can significantly influence the desired profile of *A. graveolens* essential oils, which is crucial for both medicinal and commercial applications. DT is a critical post-harvest factor affecting the preservation of essential oil quality and the overall chemical composition of plant materials.

The results indicated that shade drying led to higher yields of essential oils and certain key compounds, such as α-Phellandrene, compared to oven drying treatments. This observation aligns with previous research demonstrating that shade drying preserves the volatile components of essential oils, as high DTs can degrade delicate aromatic compounds [31]. Conversely, oven drying at 40°C and 60°C yielded varying results depending on the genotype. For instance, genotypes from Parsabad subjected to oven drying at 40°C produced the highest essential oil content, particularly in terms of Myristicin and Carvacrol. Similar findings have been reported by other researchers, indicating that lower DTs (such as 40°C) help maintain the quality of essential oilswhile providing high yields [32]. However, increasing the DT to 60°C resulted in a notable reduction observed in essential oil, particularly in genotype Ardabil. This reduction can be attributed to the heat sensitivity of certain volatile compounds, which may degrade or evaporate at higher DTs [33]. This suggests that careful control of DT is necessary to prevent the loss of valuable compounds and ensure optimal essential oil yield. The genotype × DT interactions had a substantial impact on the essential oil profile. Biplot analysis revealed that genotype × DT (CGT) combinations explained a high percentage of the variation in essential oil composition. This indicates that the interaction between genotype and DT is critical for determining the final essential oil yield and quality. The significant interaction suggests that the performance of each genotype in terms of essential oil production is influenced not only by its genetic backup but also by its post-harvest processing. For example, genotypes from Ardabil and Bushehr under shade drying conditions exhibited high essential oil yields with a favorable chemical profile, including high levels of α-Phellandrene and β-Pinene. In contrast, genotypes from Kerman subjected to oven drying at 60°C produced lower essential oil content with a less desirable chemical composition. These interactions emphasize the importance of understanding both the genetic and environmental factors influencing essential oil production. As suggested in sunflower [34] and corn [35], genotype × treatment interactions can complicate the selection of optimal genotype-treatment combinations, requiring careful analysis of each CGT to achieve the desired outcomes in terms of essential oil yield and quality. On the other hand, CGT rankings across the different characters can change depending on the interaction type, a phenomenon observed in previous studies by Sabaghnia et al. [36] in *Spinacia oleracea* and Ebrahimi et al. [37] in *Carthamus tinctorius*. These studies highlighted the complexity of selecting genotypes and treatments, emphasizing the importance of considering entry-by-tester interactions when making selection decisions. Failing to account for these interactions might lead to misleading or suboptimal genotype selection.

It facilitated a deeper understanding of the factors that influencing essential oil and its components, highlighting the importance of considering both additive and crossover interactions in the selection of genotypes and treatments for future breeding programs. The CGT × character interaction is expressed as inconsistent reactions of some characters relative to others due to CGTs rank change, known as a crossover type of interaction. Also, changes in the absolute differences between characters without rank change result in a scale change or additive type of interaction. The most important type of interaction in agriculture is due to rank change or crossover interaction, given that additive interaction does not prevent making general recommendations at the level of one factor across all levels of the other factor. The results of this research have applications for both breeding programs and post-harvest management in *A. graveolens* cultivation. Identifying genotypes that perform well under specific DTs offers valuable insights for selecting superior *A. graveolens* varieties suited to different environmental conditions. For example, genotypes from Ardabil and Bushehr, when shade-dried, exhibited the best overall performance in terms of essential oil yield and quality, making them ideal candidates for breeding programs focused on enhancing essential oil content. Additionally, these findings emphasize the role of post-harvest management practices, particularly DTs, in optimizing essential oil production. Selecting the appropriate DT based on the genotype characteristics can significantly improve the essential oil yield and quality. For instance, lower DTs (e.g., 40°C) may be more beneficial for preserving essential oil quality, while higher DTs (e.g., 60°C) should be avoided for heat-sensitive compounds. The results also demonstrate that both genetic and post-harvest factors significantly influence essential oil yield and quality. By carefully selecting genotypes and DTs, it is possible to optimize essential oil production and improve the commercial viability of *A. graveolens* as a medicinal and aromatic plant. The identified combinations of genotypes and DT are essential for the genetic improvement of *A. graveolens* and efficient post-harvest management practices. This finding is significant because essential oil quantity and quality characteristics often have low or even negative correlations in many medicinal plants, making it challenging to simultaneously enhance biological aspects [2]. Future research should explore additional environmental and agronomic factors, such as soil type, irrigation practices, and harvest timing, to further refine strategies for maximizing essential oil yield and quality in *A. graveolens* cultivation.

## 5. Conclusion

This study revealed a strong positive correlation between Myristicin and essential oil yield, highlighting the Parsabad genotype dried at 40°C as the optimal combination for maximizing these traits. Shade drying emerged as the superior post-harvest method across all genotypes, preserving essential oil quality and yield by maintaining key volatile compounds like α-Phellandrene. The significant variation in β-Pinene concentrations among genotypes underscores its potential as a key marker for future breeding and selection programs in *Anethum graveolens*. Additionally, the genotype-treatment interactions demonstrated that the Ardabil and Parsabad genotypes achieved the highest essential oil yields when oven-dried at 40°C, emphasizing the importance of tailored drying strategies based on genetic and environmental factors. These findings provide valuable insights for optimizing essential oil production through genotype selection and post-harvest management, enhancing the medicinal and commercial potential of *A. graveolens*.

## Supporting information

S1 File Supporting Information_Minimal data set. (XLSX)
